# Plasmonic Gold Nanohole Arrays for Surface-Enhanced Sum Frequency Generation Detection

**DOI:** 10.3390/nano10122557

**Published:** 2020-12-19

**Authors:** Wei Guo, Bowen Liu, Yuhan He, Enming You, Yongyan Zhang, Shengchao Huang, Jingjing Wang, Zhaohui Wang

**Affiliations:** 1 State Key Laboratory of Physical Chemistry of Solid Surfaces, MOE Key Laboratory of Spectrochemical Analysis and Instrumentation, Department of Chemistry, College of Chemistry and Chemical Engineering, Xiamen University, Xiamen 361005, China; gw2011228005@hotmail.com (W.G.); hyh4402031@163.com (Y.H.); emyou@xmu.edu.cn (E.Y.); zhangyongyan0308@163.com (Y.Z.); huangshengchao@xmu.edu.cn (S.H.); jingjing20100306@sohu.com (J.W.); 2 College of Chemistry and Chemical Engineering, Lanzhou University, Lanzhou 730000, China

**Keywords:** surface plasmon polaritons (SPP), localized surface plasmon resonance (LSPR), 4-Mercaptobenzonitrile (4-MBN), enhancement

## Abstract

Nobel metal nanohole arrays have been used extensively in chemical and biological systems because of their fascinating optical properties. Gold nanohole arrays (Au NHAs) were prepared as surface plasmon polariton (SPP) generators for the surface-enhanced sum-frequency generation (SFG) detection of 4-Mercaptobenzonitrile (4-MBN). The angle-resolved reflectance spectra revealed that the Au NHAs have three angle-dependent SPP modes and two non-dispersive localized surface plasmon resonance (LSPR) modes under different structural orientation angles (sample surface orientation). An enhancement factor of ~30 was achieved when the SPP and LSPR modes of the Au NHAs were tuned to match the incident visible (VIS) and output SFG, respectively. This multi-mode matching strategy provided flexible controls and selective spectral windows for surface-enhanced measurements, and was especially useful in nonlinear spectroscopy where more than one light beam was involved. The structural orientation- and power-dependent performance demonstrated the potential of plasmonic NHAs in SFG and other nonlinear sensing applications, and provided a promising surface molecular analysis development platform.

## 1. Introduction

Sum-frequency generation (SFG) has spurred increasing interest because of its high surface specificity, sub-monolayer sensitivity, and capability of controllable new frequency generation. As a second order nonlinear optical process, SFG is forbidden in any medium with centrosymmetry under electric-dipole approximation, but is active at the surface/interface where the centrosymmetry is broken. This makes SFG an intrinsically surface-sensitive technique, which can exclude the contributions from the bulk, and be a surgical tool to study the adsorbed species at interfaces, such as water/air interfaces [1,2,3,4], solid/air interfaces [5], protein surfaces [6,7], and electrochemical surfaces [8,9]. However, SFG is often frustrated by its high incident power and low signal flux, especially for in situ characterization and dynamic analysis of trace concentration species.

To improve the SFG signal at the interfaces, localized surface plasmon resonance (LSPR) supported on various nanostructures has been used to enhance the SFG responses of interfacial materials [10,11,12,13,14,15,16]. Baldelli et al. observed a 10^4^ times SFG enhancement of CO adsorbed on platinum nanoparticle (NP) arrays [10]. Lis et al. estimated an SFG enhancement factor (EF) of ~200 for alkanethiols adsorbed on Au nanopillars [11]. Pluchery et al. obtained an EF of 21 for the 3058 cm^−1^ thiophenol SFG band on Au NPs [12], and Barbillon et al. showed the enhanced SFG signal of two vibrational modes of thiophenol molecules at 3050 cm^−1^ and 3071 cm^−1^ on Au nanotriangles [13]. Furthermore, the plasmonic enhancements of molecular SFG vibrational signatures have been verified experimentally on Au NPs [15,16]. Although LSPR can induce strong enhancements, most of the LSPR structures are not well-defined, and small variations in the structure could lead to significant differences in the local field. For complicated in situ systems, a well-defined substrate with a remarkably large area although with a lower EF will be quite beneficial for surface analysis. 

Compared with LSPR systems, most surface plasmon polariton (SPP) nanostructures are more specific in structure, with a relatively larger uniform area [17,18]. SPPs are transverse magnetic polarized optical surface electromagnetic waves that can propagate tens to hundreds of micrometers along the interface between metal and dielectric materials [19,20,21,22]. The prism-coupled metal films in Kretschmann configuration and 2D plasmonic periodic arrays have been widely employed in the investigation of SPPs [18,23,24]. Along with its availability in large areas, SPP structures can be very useful in the spectroscopy of homogeneous surface systems. However, attempts to explore the mechanisms of SPP-driven SFG are still in a primary state. So far, there have been comparatively less attempts at the enhancement efficiency of SPP-driven SFG, such as surface roughness [24], efficient propagating configurations [25,26], and magnetization [27]. Understanding the optical performance of SPP and its relation with SFG will be helpful for the application of SPP-driven SFG surface analysis. Additionally, 2D metal grating [28] and its combination with NPs [29] have a greater electromagnetic field area and EF in surface-enhanced Raman scattering (SERS) through the coupling of LSPR and SPP. Therefore, the exploration of SFG enhancement from a nanostructure with both LSPR and SPP would be interesting and beneficial for the application of SPP-driven SFG spectroscopy.

In this work, highly uniform plasmonic gold nanohole arrays (Au NHAs) with a Q-factor of ~80 were obtained and used for surface-enhanced SFG. Au NHAs were fabricated using home-built tunable holographic lithography (THL) combined with electron beam vacuum deposition [17,30]. From angle-resolved reflectance spectroscopy, three representative SPP modes of Au NHAs were identified. The SFG enhancement of 4-Mercaptobenzonitrile (4-MBN) on Au NHAs was observed, and the influence of the structural orientation of the Au NHAs was investigated.

## 2. Materials and Methods

### 2.1. Chemicals and Materials

4-Mercaptobenzonitrile (4-MBN) was purchased from Sigma-Aldrich (Shanghai, China). SU-8 2000.5 photoresist was purchased from MicroChem (Berlin, Germany). Acetone, ethanol, sulfuric acid, hydrogen peroxide, and propylene glycol methyl ether acetate (PGMEA) were purchased from Sinopharm Chemical Reagent Co Ltd. (Shanghai, China). All of the chemicals were used without further purification.

### 2.2. Fabrication of Au NHAs

Au NHAs were fabricated with a home-built THL combined with the electron beam vacuum deposition technique [17,30]. Before the fabrication of the Au NHAs, silicon single-crystal wafers were sonicated in acetone and ethanol for 10 min successively, then soaked in piranha solution (H_2_SO_4_/H_2_O_2_ = 3:1, volume ratio) for 30 min and rinsed with deionized water. 

**Figure 1:**
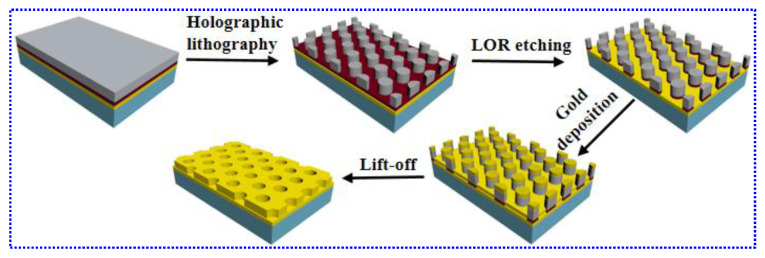
Schematic illustration of the preparation processes of the gold nanohole arrays (Au NHAs).

As illustrated in Figure 1, to fabricate Au NHAs, a 100-nm gold layer (3.5 Å/s deposition rate) was first deposited on a cleaned silicon substrate pre-coated with a 2-nm chromium adhesion layer (0.2 Å/s), followed by successive spin coating of the lift-off resist (LOR) and SU-8 photoresist with a thickness of 100 nm and 500 nm (both layer spinning for 15 s with 500 rpm, followed by 60 s with 4000 rpm), respectively. The patterned SU-8 photoresist template was prepared with THL after development. Then, the LOR layer was etched using the patterned SU-8 template to form the patterned LOR layer structure. Afterwards, a gold layer was deposited by electron beam evaporation on top of both the underlying gold layer and the patterned SU-8 photoresist. The Au NHAs were formed on the substrate after the lift-off process, i.e., the dissolution of the LOR layer with acetone, thereby removing the photoresist and top gold layer. The 100-nm gold layer was thick enough to keep the optical properties of the NHAs mainly from the gold surface, while the substrate material had no significant influence on the optical performance. 

### 2.3. Surface Characterization

The Au NHAs were rinsed with ethanol and dried with nitrogen before characterization. The morphology of the Au NHAs structures was characterized by scanning electron microscopy (SEM; S-4800, Hitachi, Tokyo, Japan) with 10 kV vacuum high voltage and 10 K magnification and atomic force microscopy (AFM; Alaph 3000, WITec, Ulm, Germany). The 512 × 512 pixel AFM was measured in tapping mode for 1 s/line scan and retrace speed and 8 µm × 8 µm scan unit (width × height). Angle-resolved reflectance was measured with a home-built system, including a spectrometer (IsoPIane SCT320, Princeton Instruments, Trenton, NJ, USA), a light source (AvaLight-DH-S-BAL, Avantes, Apeldoorn, Netherlands), and a mechanical rotation platform [17,30]. The incidence angle of the spectrometer varied from 8° to 67° with an accuracy of 0.1°.

### 2.4. Self-Assembled Monolayer (SAM) Fabrication

UV-cleaned Au NHAs samples were immersed in 1 mM 4-MBN/ethanol solution for 24 h to form SAM on the gold surfaces, which ensured full surface coverage. Prior to the SFG measurements, the 4-MBN SAM-coated Au NHAs and Au film were rinsed with ethanol to remove the residual of 4-MBN, and were dried with nitrogen.

### 2.5. SFG Measurements

The SFG spectra of the 4-MBN SAM on Au NHAs and Au film (100-nm thickness e-beam evaporated on flat glass) in a 2000–2500 cm^−1^ spectral range were collected with a home-built SFG system (Figure S1). 

The *p*-polarized VIS ($\omega_{VIS}$ = 12,440 cm^−1^, 10 cm^−1^ full width at half maximum (FWHM)) was generated from a portion of a 35-fs amplifier (Astrella, Coherent, California, USA; 6 mJ/pulse, 800 nm at 1 kHz repetition rate) output through a bandpass filter (808 nm, 3 nm FWHM, Semrock, New York, NY, USA), followed by an Etalon (800 nm, 1 nm FWHM, SLS Optics Ltd., Isle of Man, United Kingdom). The fs IR was generated from an optical parametric amplifier (TOPAS, Light Conversion, Vilnius, Lithuania). The VIS and IR ($\omega_{IR}$ = 2170 cm^−1^ and 300 cm^−1^ FWHM) were spatially and temporally overlapped on the sample surface, and the signal $\omega_{SFG}$ = $\omega_{IR}$ + $\omega_{VIS}$ radiated in the phase matching direction ($k_{SFG}\;$= $k_{IR}$ + $k_{VIS}$). The SFG signal was detected with an *i*HR 320 spectrograph (Horiba Scientific, Kyoto, Japan) using CCD (Syncerity, Horiba Scientific, Kyoto, Japan), after spectral and spatial filtering. The SFG spectra were collected in the *ppp* (SFG, VIS, and IR) polarization combination.

## 3. Results and Discussion

### 3.1. Characterization of the Au NHAs

**Figure 2:**
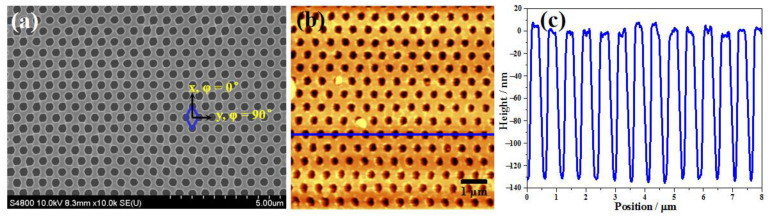
(**a**) SEM and (**b**) AFM images of the Au NHAs. (**c**) Cross-sections profile along the blue line in the AFM image.

The high-resolution SEM (Figure 2a) and AFM (Figure 2b) images demonstrated the ordered structure of the Au NHAs as a rhombus array, and each unit of the periodic structure consisted of four adjacent holes as marked in Figure 2a. Here, the structural orientation angles *ϕ* = 0° and *ϕ* = 90° were defined as being along the long axis and minor axis of the blue rhombus of the NHAs in Figure 2a. The average diameter of the holes was 323.0 ± 18.7 nm, and the periods of the Au NHAs nanostructure were *P*_1_ = 1025.3 ± 7.4 nm and *P*_2_ = 587.5 ± 12.2 nm (center to center) in the *x*- and *y*-directions from the SEM and AFM height profile (Figure 2c), respectively. The AFM height profile extracted along the blue line in Figure 2b revealed that the depth of the holes was approximately 130 nm, a bit deeper than the nominal deposition thickness of 100 nm, which was possibly caused by the shadowing effect of the electron-beam evaporation. It is worth mentioning that no significant defect was observed from the SEM and AFM images. Therefore, the present method provided a selectable route for the fabrication of high-quality metal NHAs.

### 3.2. Optical Properties of the Au NHAs

**Figure 3:**
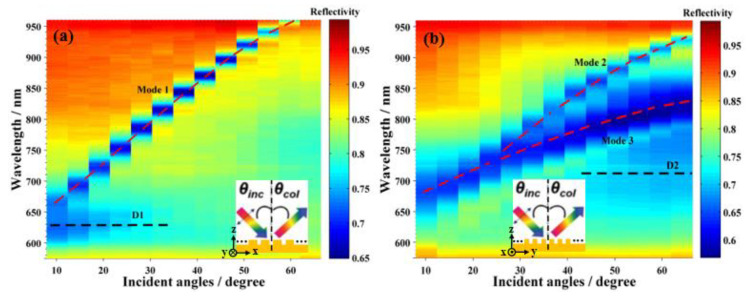
Angle-resolved reflection spectrum of the Au NHAs at different structural orientation angles (**a**) *ϕ* = 0° and (**b**) *ϕ* = 90°. Insets are the optical measurement configuration.

To investigate the optical properties of the Au NHAs, the reflectance spectra of the Au NHAs at different structural orientation angles (*ϕ*) and excitation-collection angles (*θ*) with a *p*-polarized incident light were measured as shown in Figure 3. As depicted in Figure 3, three angle-dependent (marked with red dashed lines, named Modes 1–3) and two angle-independent (marked with black dashed lines, named D1 and D2) dips in the visible and near-infrared region were observed. A narrow peak around 962 nm for *ϕ* = 0° (Mode 1, FWHM 12.5 nm) and two peaks around 882 (Mode 2, FWHM 12.2 nm) and 828.5 nm (Mode 3, FWHM 40.2 nm) for *ϕ* = 90° were assigned to the SPP of the periodic Au NHAs, which were shifted with incident angles (Figure 3 and Table S1) [17,30]. The weak and incident angle independent 630 nm (D1) and 710 nm (D2) were caused by the LSPR of the Au holes [31,32]. The corresponding quality factors *Q* = *λ_0_/Δλ* (where *λ_0_* was the resonant wavelength and *Δλ* was the corresponding FWHM) were 77, 76, and 21 for Modes 1–3 of the Au NHAs, respectively. What called for special attention was that such a narrow bandwidth and high *Q*-factor SPP modes of Au NHAs, which were functional in a large incident angle range and broad spectral range, could be excellent candidates for SPP sensing [17].

### 3.3. Plasmonic Enhanced SFG of 4-MBN on the Au NHAs

**Figure 4:**
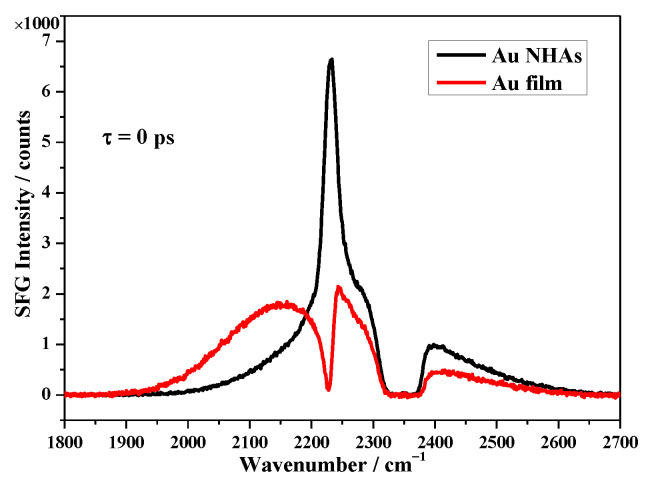
SFG spectra of 4-MBN on Au NHAs (black) at *ϕ* = 90° and flat gold film (red) at ~57° incident angle (with 1 mW VIS, 5 mW IR, and 30 s acquisition time).

Figure 4 displays the SFG spectra of 4-MBN on Au NHAs (*ϕ* = 90°) and a smooth flat Au film surface in the −C≡N stretching range of around 2230 cm^−1^. The SFG spectra consisted of narrow resonant SFG bands from the 4-MBN molecules superimposed on the broadband Au non-resonant features, the resonant SFG band appeared as a peak in the Au NHAs spectra and as a dip/valley in the flat Au film spectrum at a delay time of *τ* = 0 ps (time difference between the IR and VIS pulse arriving at the surface). The measured CN SFG band position was consistent with previous studies (irrespective of the CO_2_ absorption around 2350 cm^−1^) [33,34].

The SFG signal was the coherent addition of the non-resonant response of the substrate (insensitive to IR wavelength) and the resonant vibrational contributions of the surface molecules, and could be modeled with Equation (1):

(1) $$I_{SFG}\propto {\left|\chi_{NR}^{\left(2\right)}+\chi_{R}^{\left(2\right)}\right|}^{2}E_{IR}^{2}={\left|A_{NR}+\sum_{n}\frac{A_{n}\Gamma_{n}e^{i\Phi_{n}}}{\omega_{IR}-\omega_{n}+i\Gamma_{n}}\right|}^{2}e^{-\frac{{\left(\omega_{IR}-\omega_{IR,0}\right)}^{2}}{\Gamma_{0}^{2}}},$$

where $\chi_{NR}^{\left(2\right)}$ and $\chi_{R}^{\left(2\right)}$ are the non-resonant and resonant second order nonlinear susceptibilities; *A_n_*, *ω_n_*, and *Γ_n_* are the amplitude, resonance frequency, and damping constant (homogeneous line width) of the transition of the *n*th vibration, $\omega_{IR}$ and $\omega_{IR,0}$ were the vibrational frequency and center frequency of the IR pulse, respectively; and *Γ_0_* is the damping constant of the IR pulse. *A_NR_* is the amplitude of the non-resonant, and *Φ_n_* is the phase difference between the resonant and non-resonant. The line shape of the SFG resonances was likely the consequence of joint contributions of *A_n_,* *A_NR_,* and *Φ_n._*

The fitting parameters for Equation (1) are summarized in Table 1. The *A_NR_* of the Au NHAs and Au film were very close. The *Γ_n_* of the resonant band on Au NHAs was about 1.6 times that on the flat Au surface, which meant faster vibrational dephasing on Au NHAs [35]. As 4-MBN molecules anchor to the Au surface through Au-S bonding, the orientations of the 4-MBN with respect to the Au normal surface were the same or very similar on both the Au NHAs and flat Au films. In fact, there was only a 11° difference in the relative phase, *Φ_n_* (between resonant and non-resonant), 277.7° for the Au NHAs, and 288.7° for the Au film. However, this phase difference could not turn the overall line shape of the SFG spectra from a peak into a valley. Spontaneously, the resonant amplitudes (*A_n_*) were considered to be the major factor for the overall SFG spectral line shape changes in Figure 4. The phase difference was introduced by the 4-MBN near the edges of the NHAs holes with slightly different orientations [32,36]. In addition, the ratio of, *A_n_*, for the Au NHAs and Au film is 2.8, which corresponded to an eight times enhancement of the SFG signal intensity, i.e., EF is the ratio of the resonant SFG band intensity on Au NHAs (SE-SFG) and flat Au surface (normal SFG).

**Figure 5:**
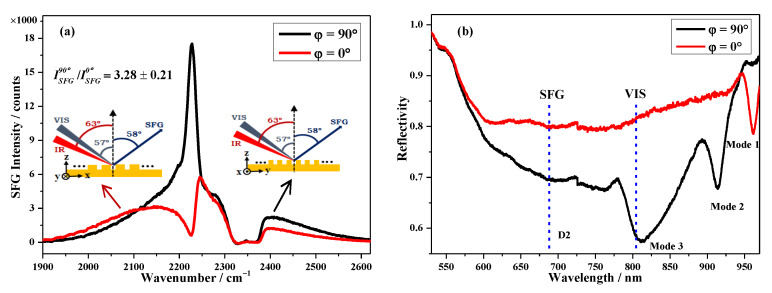
(**a**) SFG spectra of 4-Mercaptobenzonitrile (4-MBN) on Au NHAs with different structural orientation angles (with 1 mW VIS, 5 mW IR, and 60 s acquisition time). Insets are the SFG optical configurations. (**b**) Structural orientation dependent reflectance spectra of Au NHAs at a 57.4° incident angle.

In order to evaluate the effectiveness of the SPP modes, the SFG spectra of 4-MBN on the Au NHAs at structural orientation angles of *ϕ* = 0° and *ϕ* = 90° were measured and are shown in Figure 5a. Although the SFG spectrum of *ϕ* = 0° was similar to that on the flat Au film (red spectrum in Figure 4), the resonant band amplitude was enhanced with an EF of ~3. The ratio of the −C≡N SFG band intensities of *ϕ* = 90° and 0°, $I_{SFG}^{90^{{}^{\circ}}}/I_{SFG}^{0^{{}^{\circ}}}$, was 3.26 ± 0.19 from the fitting result of Equation (1). The incident angles of VIS and IR were 57° and 63°, as shown in Figure 5a, respectively. The SFG signal was at the phase-matching angle of *θ_SFG_* = 58° with respect to the normal surface. Figure 5b shows the structural orientation angle dependent reflectance spectra of the Au NHAs with a 57.4° incident angle. With the 57.4° incident angle, the VIS (804 nm) and SFG (682 nm; marked with blue dashed lines in Figure 5b) were resonant with Mode 3 and D2 of the Au NHAs, respectively. The IR (4400 nm) was far away from the SPR or LSPR of the Au NHAs, and its enhancement was negligible. It has been well-documented that the electromagnetic field enhancement introduced by the SPR/LSPR of metallic nanostructures is the predominant mechanism in SERS [37,38,39], TERS [40], and surface-enhanced fluorescence spectroscopy [41]. Extinction intensities, *Qe*, was a good measurement of the local SPR field. Similar to SERS [42], larger SFG signals were detected when both the incident VIS and the output SFG matched with the SPR, and its EF was proportional to the extinction intensity [43,44].

(2) $$\mathrm{EF}\propto Q_{e}\left(\lambda_{\mathrm{VIS}}\right)Q_{e}\left(\lambda_{\mathrm{SFG}}\right).$$

The extinction intensity, *Qe,* was proportional to the absorbance (*A*) of the NHAs ($Q_{e}\propto A$), which could be obtained directly from the reflectance (*R*), that is, *A* = 1 − *R* [45,46]. Because both the incident VIS and the output SFG fields could be enhanced by the SPR or SPP, the absorbance at each corresponding wavelength were used to estimate the EF difference for 90° and 0° structure orientations.

(3) $$\frac{EF^{90^{{}^{\circ}}}}{EF^{0^{{}^{\circ}}}}=\frac{A_{VIS}^{90^{{}^{\circ}}}\times A_{SFG}^{90^{{}^{\circ}}}}{A_{VIS}^{0^{{}^{\circ}}}\times A_{SFG}^{0^{{}^{\circ}}}}.$$

Using the reflectance values from Figure 5b, $A_{VIS}^{90^{{}^{\circ}}}=1-0.58=0.42,\;A_{SFG}^{90^{{}^{\circ}}}=1-0.70=0.30,A_{VIS}^{0^{{}^{\circ}}}=1-0.81=0.19,\;\mathrm{and}\;A_{SFG}^{0^{{}^{\circ}}}=1-0.80=0.20,$ and putting these values into Equation (3), the ratio was calculated as 3.32, which was very close to the 3.26 measured by $I_{SFG}^{90^{{}^{\circ}}}/I_{SFG}^{0^{{}^{\circ}}}$ in Figure 5a. This result revealed that exciting along the minor axis of the rhombus of the Au NHAs (*ϕ* = 90°) could obtain a more remarkable SFG enhancement, and both the SPP and LSPR of the Au NHAs cooperatively contributed to SFG enhancement.

**Figure 6:**
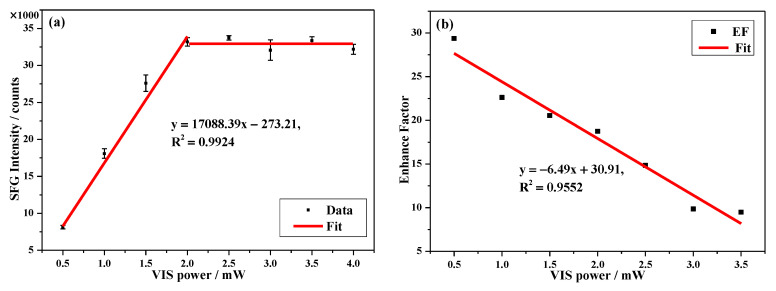
VIS power-dependent (**a**) SFG intensity of 4-MBN and (**b**) SFG enhancement factor of the Au NHAs at *τ* = 1 ps (with 5 mW IR and 60 s acquisition time).

The enhanced SFG intensity was saturated around 2 mW (2 µJ/pulse) with *ϕ* = 90°, as shown by the VIS power-dependent SFG intensity (*τ* = 1 ps) in Figure 6a. It was most likely that a high VIS pulse energy caused sample damage, especially in the hotspots. Figure 6b displays the power-dependent EF of the Au NHAs, which exhibited about a 30-fold SFG enhancement at low VIS powers. Consequently, the decrease of EF with the incident power implied that the SPP damping was power-dependent, and the VIS power could be further optimized. Potentially, it was possible to achieve a higher EF with even lower incident powers. Additionally, only a very small fraction of the anchored 4-MBN molecules was located in the SPP region. Fang et al. showed the hotspot of an Ag film on nanospheres comprised <1% of the total surface molecules, but contributed ~70% of the overall SERS signal [47]. Therefore, the actual SFG EF of the Au NHAs should be much greater than the measured value of ~30 under the consideration of the hotspot area. Further optimization of NHAs with a high *Q*-factor, and tuning its multiple SPP modes to match both the VIS and SFG, likely could be generalized to other nonlinear methods, and would greatly improve the enhancement capability. These results demonstrated the promising potential of Au NHAs in SFG enhancement and related applications in molecular sensing and analysis.

## 4. Conclusions

In this work, the optical properties of THL fabricated Au NHAs were characterized with morphology and reflectance spectroscopy. The surface enhancement of the nonlinear SFG process through multi-coupling with the SPP and LSPR was evaluated with a SFG spectroscopy of 4-MBN on Au NHAs. The angle-resolved reflectance spectra showed that the Au NHAs with a Q-factor of ~80 had three typical SPP modes and two non-dispersive LSPR modes. By exciting along the minor axis of the rhombus of the Au NHAs (ϕ = 90°), a remarkable SFG enhancement was obtained through the incident (VIS-SPP) and output (SFG-LSPR) matching with the surface plasmonic field simultaneously. The multi-mode matching strategy provided flexible control and selective spectral windows for surface-enhanced SFG, and was especially useful in nonlinear spectroscopy where more than one light beams were involved. The SFG EF was inversely correlated with the incident VIS pulse energy, which suggested a greater enhancement potential of Au NHAs with optimal incident pulse energies. These results indicated that Au NHAs has a promising potential in SFG enhancement and related applications.

## Figures and Tables

**Table 1:** Parameters of the SFG spectra on Au NHAs and flat Au film.

Sample	*A_NR_*	*A_n_*	*ω_n_*/cm^−1^	*Γ_n_*/cm^−1^	ϕ*_n_*/°
Au NHAs	37.0	119.3	2229	13.5	277.7
Au film	44.4	42.4	2230	8.2	288.7

## Supplementary Materials

The following are available online at https://www.mdpi.com/2079-4991/10/12/2557/s1. Figure S1: Schematic of broadband SFG (BB-SFG) experimental setup. Figure S2: Determination of the FWHM value of the SPP modes. Table S1: Dip positions and FWHM values of the SPP modes of the Au NHAs.
