# Supplementary material for: Plasmonic Gold Nanohole Arrays for Surface-Enhanced Sum Frequency Generation Detection
Source: Nanomaterials (Basel). 2020 Dec 19;10(12):2557. doi: 10.3390/nano10122557 (PMC7766786; doi:10.3390/nano10122557)
Supplement: Supplementary file 1 [file nanomaterials-10-02557-s001.pdf]

nanomaterials

Supplementary Materials

Plasmonic Gold Nanohole Arrays for Surface-Enhanced Sum Frequency Generation Detection

Wei Guo, Bowen Liu, Yuhan He, Enming You, Yongyan Zhang, Shengchao Huang, Jingjing Wang and Zhaohui Wang

S1. SFG and Surface-Enhanced SFG (SE-SFG)

Sum-frequency generation (SFG) is a second-order nonlinear spectroscopic technique. When the incident laser beams of VIS and IR are spatially and temporally overlapped on the sample surface, the SFG signal $\omega_{SFG} = \omega_{IR} + \omega_{VIS}$ radiates at the phase matching direction ($k_{SFG} = k_{IR} + k_{VIS}$). SFG is surface/interface selective under dipolar approximation. Through SFG spectroscopy, the surface molecular structure, orientation, packing, and dynamics (ultrashort pulses used in SFG) may be explored. The theory of SFG has been profoundly expounded in the literature [1–3]. If the electric field of the incident and/or output radiation is coupled to the localized surface plasmon resonance (LSPR)/ surface plasmon polariton (SPP) of the interfacial materials, the SFG signal will be enhanced, namely surface-enhanced SFG (SE-SFG), which can be realized similarly to SERS. There are few major aspects worth mentioning in SE-SFG: (1) SFG is a coherent optical process, the role of two incident beams needs to be investigated; (2) ultrashort laser pulse has much higher energy flux than a continuum laser source, which may induce extra nonlinear effects; and (3) photo-induced damping will increase electron–electron scattering, and reduce the dephasing time and EF. Lis et al. have reviewed the application of SE-SFG in the plasmonic nanomaterials [3]. Recently, Busson et al. summarized SE-SFG [4] and the significant role of hotspot in SE-SFG [5]. He et al. did pioneering research on shell-isolated-nanoparticle-enhanced SFG (SHINE-SFG) and proposed a new mechanism of SE-SFG, i.e., the nonlinear coupling of SHINE-SFG with difference frequency generation (DFG) [6].

S2. SFG Experimental Setup

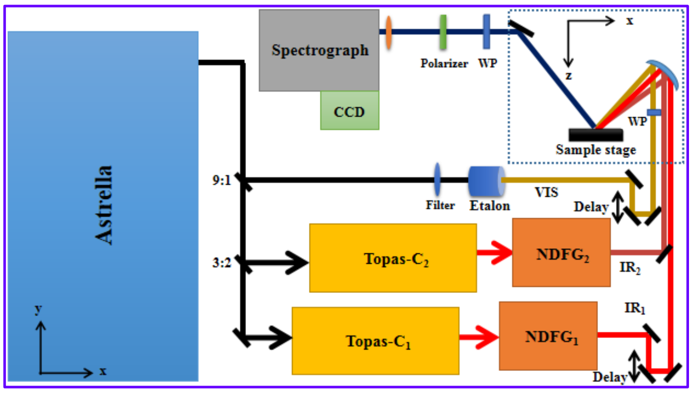

Figure S1. Schematic of broadband SFG (BB-SFG) experimental setup.

The SFG measurements were performed through reflection geometry, as shown in Figure S1. The light sources were generated with a 35 fs amplifier (Astrella, Coherent, Santa Clara, CA, USA), and a 6 mJ/pulse, centered at 800 nm at 1 kHz repetition rate. A small portion of the amplifier output (10%) was passed through a narrowband filter (808 nm, 3 nm full width at half maximum (FWHM), Semrock, New York, NY, USA) and an Etalon (800 nm, 1 nm FWHM, SLS Optics Ltd., Isle of Man, United Kingdom) to generate the VIS beam. The rest of the amplifier output was used to generate the IR with a commercial optical parametric amplifier (TOPAS, Light Conversion, Vilnius, Lithuania) and non-colinear difference-frequency generation (NDFG). As illustrated in Figure S1, the VIS and IR beams are in the same plane with incident angles of 57° and 63° with respect to the surface normal,

and were focused on the sample with the spot diameter of 260 μm and 500 μm, respectively. The incident energies of the IR and VIS at the sample were 5 μJ/pulse and 1 μJ/pulse, resepectviely, if not otherwise specified.

S3. FWHM Values of the SPP Modes

The reflectance value at the dip is denoted as R_1, and that at the left/right shoulder is denoted as R_2. The FWHM of the SPP mode is then taken as the reflectance value of $(R_1 + R_2)/2$, as indicated with the double-arrow line in Figure S2 [7]. The calculated FWHM values and the dip positions of the SPP modes are summarized in Table S1.

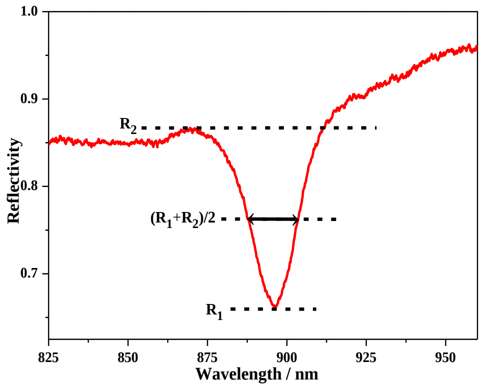

Figure S2. Determination of the FWHM value of the surface plasmon polariton (SPP) modes.

Table S1. Dip positions and FWHM values of the SPP modes of the gold nanohole arrays (Au NHAs).

Incident Angle (°)	Mode 1 (nm)	FWHM 1 (nm)	Mode 2 (nm)	FWHM 2 (nm)	Mode 3 (nm)	FWHM 3 (nm)
7.9	663.5	--	--	--	682.9	--
12.4	692.9	22.0	--	--	692.5	--
16.9	722.1	24.4	708.4	--	708.4	62.2
21.4	753.2	25.4	727.7	--	727.7	62.4
25.9	785.2	25.4	753.9	--	743.4	71.9
30.4	814.4	21.9	789.1	14.0	750.4	79.0
34.9	842.8	20.2	815.4	13.1	757.5	64.8
39.4	870.3	16.7	842.6	20.2	754.0	45.6
43.9	896.0	16.6	861.9	14.1	786.4	43.8
48.4	919.8	15.7	882.0	12.2	798.7	42.1
52.9	941.3	13.9	897.7	14.1	805.7	54.3
57.4	961.6	12.5	913.6	14.1	812.8	52.8
61.9	--	--	927.6	14.0	823.2	50.8
66.4	--	--	938.1	12.4	828.5	40.2

References

1. Shah, S.A.; Baldelli, S. Chemical Imaging of Surfaces with Sum Frequency Generation Vibrational Spectroscopy. *Acc. Chem. Res.* **2020**, *53*, 1139–1150.
2. Tang, F.; Ohto, T.; Sun, S.; Rouxel, J.R.; Imoto, S.; Backus, E.H.G.; Mukamel, S.; Bonn, M.; Nagata, Y. Molecular Structure and Modeling of Water-Air and Ice-Air Interfaces Monitored by Sum-Frequency Generation. *Chem. Rev.* **2020**, *120*, 3633–3667.

3. Lis, D.; Cecchet, F. Localized surface plasmon resonances in nanostructures to enhance nonlinear vibrational spectroscopies: towards an astonishing molecular sensitivity. *Beilstein J. Nanotech.* **2014**, *5*, 2275–2292.
4. Humbert, C.; Noblet, T.; Dalstein, L.; Busson, B.; Barbillon, G. Sum-Frequency Generation Spectroscopy of Plasmonic Nanomaterials: A Review. *Materials* **2019**, *12*, 836.
5. Dalstein, L.; Humbert, C.; Ben Haddada, M.; Boujday, S.; Barbillon, G.; Busson, B. The Prevailing Role of Hotspots in Plasmon-Enhanced Sum-Frequency Generation Spectroscopy. *J. Phys. Chem. Lett.* **2019**, *10*, 7706–7711.
6. He, Y.; Ren, H.; You, E.M.; Radjenovic, P.M.; Sun, S.G.; Tian, Z.Q.; Li, J.F.; Wang, Z. Polarization- and Wavelength-Dependent Shell-Isolated-Nanoparticle-Enhanced Sum-Frequency Generation with High Sensitivity. *Phys. Rev. Lett.* **2020**, *125*, 047401.
7. Shen, Y.; Zhou, J.; Liu, T.; Tao, Y.; Jiang, R.; Liu, M.; Xiao, G.; Zhu, J.; Zhou, Z.K.; Wang, X.; Jin, C.; Wang, J. Plasmonic gold mushroom arrays with refractive index sensing figures of merit approaching the theoretical limit. *Nat. Commun.* **2013**, *4*, 2381.

© 2020 by the authors. Submitted for possible open access publication under the terms and conditions of the Creative Commons Attribution (CC BY) license (http://creativecommons.org/licenses/by/4.0/).
